# Evaluation of MicroRNA Expression in Patient Bone Marrow Aspirate Slides

**DOI:** 10.1371/journal.pone.0042951

**Published:** 2012-08-13

**Authors:** Leah Morenos, Richard Saffery, Francoise Mechinaud, David Ashley, Ngaire Elwood, Jeffrey M. Craig, Nicholas C. Wong

**Affiliations:** 1 Cancer and Disease Epigenetics, Murdoch Children’s Research Institute, Melbourne, Victoria, Australia; 2 Department of Paediatrics, The University of Melbourne, Melbourne, Victoria, Australia; 3 Children’s Cancer Centre, Royal Children’s Hospital, Melbourne, Victoria, Australia; 4 Early Life Epigenetics, Murdoch Children’s Research Institute, Melbourne, Victoria, Australia; 5 Andrew Love Cancer Centre, Deakin University, Geelong, Victoria, Australia; 6 BMDI Cord Blood Bank, Murdoch Childrens Research Institute, Melbourne, Victoria, Australia; University of Minnesota Medical School, United States of America

## Abstract

Like formalin fixed paraffin embedded (FFPE) tissues, archived bone marrow aspirate slides are an abundant and untapped resource of biospecimens that could enable retrospective molecular studies of disease. Historically, RNA obtained from slides is limited in utility because of their low quality and highly fragmented nature. MicroRNAs are small (≈22 nt) non-coding RNA that regulate gene expression, and are speculated to preserve well in FFPE tissue. Here we investigate the use of archived bone marrow aspirate slides for miRNA expression analysis in paediatric leukaemia. After determining the optimal method of miRNA extraction, we used TaqMan qRT-PCR to identify reference miRNA for normalisation of other miRNA species. We found hsa-miR-16 and hsa-miR-26b to be the most stably expressed between lymphoblastoid cell lines, primary bone marrow aspirates and archived samples. We found the average fold change in expression of hsa-miR-26b and two miRNA reportedly dysregulated in leukaemia (hsa-miR-128a, hsa-miR-223) was <0.5 between matching archived slide and bone marrow aspirates. Differential expression of hsa-miR-128a and hsa-miR-223 was observed between leukaemic and non-leukaemic bone marrow from archived slides or flash frozen bone marrow. The demonstration that archived bone marrow aspirate slides can be utilized for miRNA expression studies offers tremendous potential for future investigations into the role miRNA play in the development and long term outcome of hematologic, as well as non-hematologic, diseases.

## Introduction

MicroRNA (miRNA) are a functionally-important class of small, non-protein coding RNA that control gene expression post-transcriptionally. This is an important layer of gene regulation within all eukaryotic cells, and when perturbed can give rise to disease [1], [2], [3]. At approximately 22 nucleotides long, the mature miRNA is combined with RISC (RNA Induced Silencing Complex) and Argonaute proteins to inhibit specific target messenger RNA (mRNA) through base pair recognition [4], [5]. *In silico* analysis predicts over 1,000 miRNA genes within the human genome [4], with each miRNA having the potential to regulate as many as 200 targets, equalling over 30% of all human genes [6], [7]. Aberrant expression of miRNAs has also been linked to critical gene pathways in cancer development and progression.

Fresh tissue samples are the typically preferred source of DNA, RNA and protein for disease analysis [2]. However with a current absence of concomitant biobanking of disease specimens, researchers are beginning to turn to alternative sources of biospecimens such as those archived as part of routine clinical care. Pathology and histology laboratories worldwide contain large stocks of archived samples with potential utility for molecular analysis [3] such as formalin-fixed paraffin-embedded (FFPE) tissues and glass slide smears for haematological disorders. Importantly, material archived as part of clinical care is usually associated with extensive clinicopathological data, potentially allowing for retrospective examination of specific molecular markers and clinical disease associations [3]. Much interest has therefore been placed on exploring the utility of various archived biospecimens for molecular analyses.

In recent years the protocols for the extraction of DNA, mRNA, miRNA and proteins from archived material have improved enormously [2], [8], [9]. Previous reports have demonstrated the isolation of PCR-amplifiable DNA and RNA from archival unstained bone marrow slides [10], [11], Giemsa-stained bone marrow and peripheral blood smears [12], [13], [14], stored whole peripheral blood [15] and dried blood Guthrie spots [16]. However, the isolation of sufficient amounts of DNA and RNA for disease interrogation from FFPE samples remains challenging [2]. Much attention therefore has been given to the possibility of using archived FFPE samples for miRNA interrogation. It is believed that miRNA are less susceptible to fragmentation and degradation because of their small size [17], [18]. Many studies have demonstrated a good correlation between miRNA expression in FFPE and matched fresh-frozen tissues [3], [8], [18], [19], with more stable and consistent expression of miRNA in FFPE for quantitative Real-Time PCR [9], [17], [20], microarray [19], [21] and deep sequencing analysis [22].

Unlike FFPE samples, the utility of archived bone marrow film slides for miRNA expression studies has yet to be elucidated. The current study is an exploratory investigation into the miRNA expression relationship between archived slides and their matched fresh-frozen tissue, using paediatric acute leukaemia samples (bone marrow from acute lymphoblastic [ALL] and acute myeloid [AML] patients).

We have investigated optimal miRNA extraction methods for use with archived bone marrow aspirate slides and appropriate for miRNA expression analysis. Our approach in investigating miRNA expression on archived bone marrow smears could equally be applied to study other hematologic diseases where bone marrow and blood films are routinely archived. We can now exploit a largely untapped source of samples available in most pathology laboratories worldwide.

## Results and Discussion

It has been shown in many studies that archived unstained patient slide smear samples return good quality, amplifiable DNA [10], [11], [12], [13], but can have degraded total RNA [17], [18]. The innate small size of miRNAs however has been hypothesized to decrease their chances of degradation during tissue processing [17], [18], and mature miRNAs may be less susceptible to fragmentation due to their close association with the RISC protein complex [1], [19], [21]. Here we have investigated the most efficient RNA extraction methods which have applicability for miRNA expression analysis; however a sufficient internal Reference gene for normalization of our experimental expression data needed to be first established.

### Establishment of Reference Genes for Archived Bone Marrow Samples

**Figure 1:**
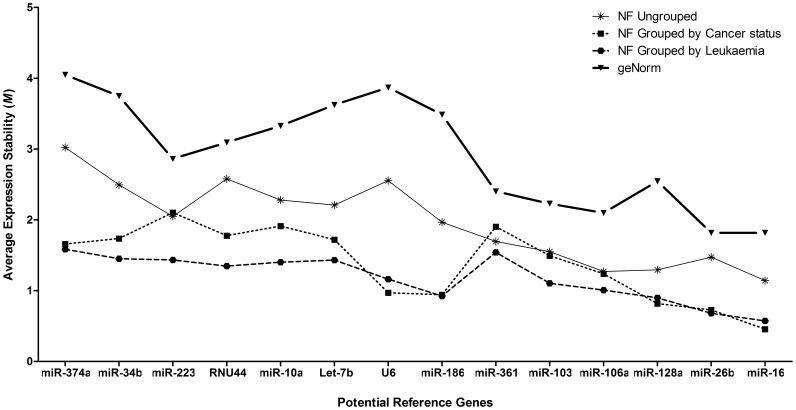
The stability of miRNA species expression in hematopoietic samples. The stability of miRNA species expression using 41 samples of leukaemic and non-leukaemic cell lines, paediatric archived bone marrow aspirate slides (Table S1), and 14 potential miRNA candidates (Table S2). geNorm and NormFinder (NF) were used to calculate the expression stability values for each miRNA tested. The most ‘stably’ expressed gene in a sample group is that with an average expression stability value (*M*) closest to zero. The most stably expressed miRNA were; NF Ungrouped: hsa-miR-16 *M* = 1.143; NF Grouped by Cancer Status: hsa-miR-16 *M* = 0.457; NF Grouped by Leukaemia subtype: hsa-miR-16 *M* = 0.573; geNorm: hsa-miR-16 and hsa-miR-26b are equal at *M* = 1.817.

The accuracy of miRNA expression analysis is dependent on the proper choice of endogenous controls, whereby inappropriate normalization may lead to incorrect conclusions [23], [24]. A number of samples were used to identify internal reference miRNAs that remain stably expressed, here for leukaemia and hematopoietic diseases (Table S1). Model cell lines (n = 14) and archived aspirate slides (n = 27) were used to evaluate the expression stability of 14 putative miRNA reference genes utilizing geNorm [25] and NormFinder (NF) [26] software (Table S2). The best reference candidates identified here were hsa-miR-16 and hsa-miR-26b (Figure 1). The expression of these miRNA are the most consistent across the tissue types, disease states and processing variables of the samples in our set (NF Ungrouped: hsa-miR-16 *M* = 1.14; NF Grouped by cancer status: hsa-miR-16 *M* = 0.46 & hsa-miR-26b *M* = 0.73; NF Grouped by Leukaemia subtype: hsa-miR-16 *M* = 0.57 & hsa-miR-26b *M* = 0.68; geNorm: hsa-miR-16 & hsa-miR-26b are equal at *M* = 1.82). hsa-miR-16 and hsa-miR-26b have been previously reported as stable in various human tissues and cell lines [23], [27], in prostate and breast cancer models [28] as well as in plasma and serum [29].

### Optimization of miRNA Extraction from Archived Bone Marrow Samples

**Figure 2:**
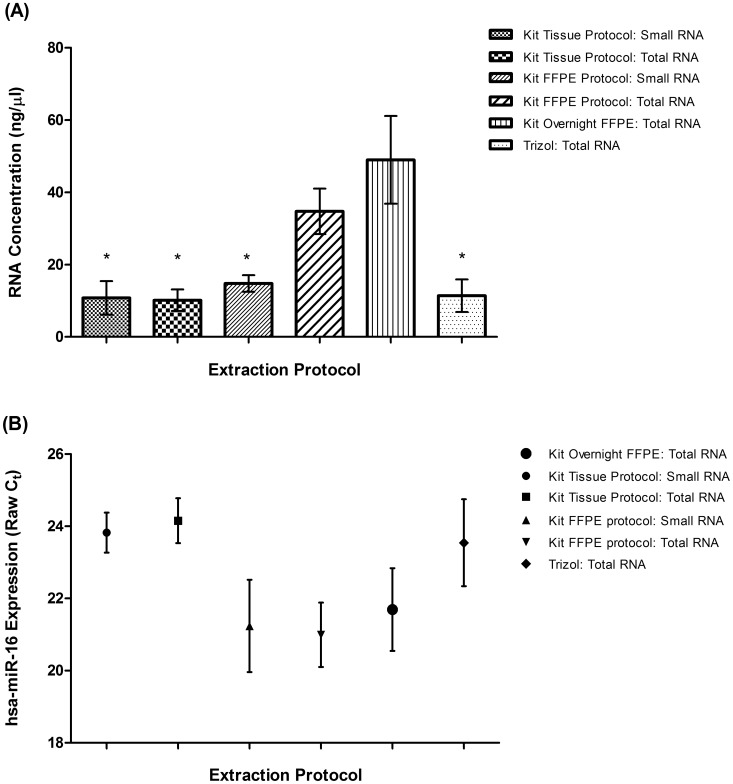
A comparison of RNA extraction methods for the evaluation of RNA concentration (ng/µl) and miRNA expression from archived bone marrow aspirate slides. The comparison of miRNA extraction methods from matched unstained archived bone marrow smears (n = 6 or n = 8 patients; Table S3). Six different methods were used based on traditional TRIzol or using the Roche High Pure miRNA Column extraction kit (Table 1). (**A**) **Comparison of Total RNA yield from experimental extraction protocols**. The average RNA concentration was measured by NanoDrop (ng/µl ±s.e.m.). Kit Total RNA extraction methods with a Proteinase K digestion (‘FFPE protocol’) are higher in RNA yield compared to kit methods without a digestion step (‘Tissue protocol’), or when extracting small RNA only. These methods are also significantly better than TRIzol extractions. The archived slide extraction method with the highest RNA yield was the Roche kit ‘isolation from FFPE Tissue’ Total RNA extraction with an overnight Proteinase K digestion. * Indicates RNA concentration is significantly different to the Kit Overnight FFPE: Total RNA protocol. (**B**) **Comparison of hsa-miR-16 expression from experimental extraction protocols**. The average expression of our reverence gene (hsa-miR-16) measured by qRT-PCR (raw C_t_ ±s.e.m.). Raw C_t_ value tracks with total RNA yield, whereby as the concentration of RNA increases the C_t_ value decreases. This analysis shows extractions with a Proteinase K digestion return a lower C_t_ value compared to extractions without a digestion step. TRIzol extractions also return a higher C_t_ value than the Proteinase K digestion protocols.

To optimize RNA extraction from archived slide smear samples several methods were trialled utilizing the Roche High Pure miRNA isolation kit and TRIzol extraction. Unstained patient smear samples from nine individuals (leukaemic and non-leukaemic; Table S3) were used to evaluate the quality of six slide miRNA extraction protocols (Table 1). We found here, as with others, that longer Proteinase K digestion significantly increased RNA yield (Figure 2A) [30], [31], [32]. The Roche overnight ‘isolation of FFPE tissue’ protocol produced the highest total RNA yield in this study (Mean 48.97 ng/µl (±12.15 s.e.m.)). An equivalent extraction protocol with a 3 hour digestion produced slightly lower RNA yield for total RNA (Mean 34.7 ng/µl (±6.29 s.e.m.)), and significantly lower yield for small RNA extracts (Mean 14.77 ng/µl (±2.28 s.e.m.) Wilcoxon 2-tailed T-test p = 0.031). The ‘isolation of tissue’ protocols (homogenization of samples only) were the least effective for RNA recovery, and returned significantly lower yields then the FFPE overnight protocol (Mean Small RNA: 10.78 ng/µl (±4.66 s.e.m.) Wilcoxon 2-tailed T-test p = 0.031; Total RNA: 10.14 ng/µl (±2.97 s.e.m.) Wilcoxon 2-tailed T-test p = 0.031).

**Table 1:** Modified protocols used for the extraction of RNA from archived bone marrow aspirate slides.

Method Name	Product Used	Protocol	Slide Scraping	Digestion	RNA extraction
***1***: Kit Tissue: Small RNA	Roche High Pure miRNA Extraction Kit	‘Isolation from Tissue’	300 µl Lysis buffer	Homogenization Only	Small RNA Only
***2***: Kit Tissue: Total RNA	Roche High Pure miRNA Extraction Kit	‘Isolation from Tissue’	300 µl Lysis buffer	Homogenization Only	Total RNA
***3***: Kit FFPE: Small RNA	Roche High Pure miRNA Extraction Kit	‘Isolation from FFPE Tissue’	200 µl Lysis buffer	Proteinase K 3 hrs	Small RNA Only
***4***: Kit FFPE: Total RNA	Roche High Pure miRNA Extraction Kit	‘Isolation from FFPE Tissue’	200 µl Lysis buffer	Proteinase K 3 hrs	Total RNA
***5***: Kit Overnight FFPE: Total RNA	Roche High Pure miRNA Extraction Kit	‘Isolation from FFPE Tissue’	200 µl Lysis buffer	Proteinase K Overnight	Total RNA
***6***: TRIzol: Total RNA	TRIzol	‘Total RNA Isolation’	400 µl TRIzol	Homogenization Only	Total RNA

The modified extraction protocols used in this study for the extraction of total RNA and small RNA from unstained archived bone marrow aspirate slides. Two main methods were used, the Roche High Pure miRNA Isolation Kit and TRIzol. We followed two provided extraction protocols for the kit; ‘miRNA isolation from tissue’, and ‘miRNA isolation from FFPE tissue’. The methods provided by the manufacturers were modified to include a slide scraping step, and a digestion/homogenization step to break down the slide material. The kit allows for the extraction of total RNA as well as small RNA only; both these methods were trialled here; samples listed in Table S3.

To confirm these extraction techniques were suitable for miRNA expression analysis, quantitative RT-PCR was used to measure the expression of our identified reference miRNA (hsa-miR-16) (Figure 2B). The methods of extraction utilizing sample homogenization had the highest C_t_ results, which correspond inversely to the lowest expression levels of hsa-miR-16, and the lowest RNA yield. Values were improved by the addition of Proteinase K where the kit ‘isolation of FFPE tissue’ total RNA protocols returned the lowest C_t_, therefore the highest miRNA expression, and higher RNA yield (Total RNA 3 hr digestion 20.99±0.89 s.e.m; Total RNA overnight digestion 21.69±1.15 s.e.m). The TRIzol technique however provided both a significantly lower total RNA yield in comparison to the overnight kit protocol (Mean 11.37 ng/µl (±4.51 s.e.m.) Wilcoxon 2-tailed T-test p = 0.008), as well as a higher C_t_ value (23.54±1.2 s.e.m) compared to all other protocols. TRIzol extractions of total RNA from archived slides may potentially cause the loss of small RNA species by inefficient RNA precipitation compared to column-based isolation methods. It has been recently suggested that loss of small RNA species in TRIzol extractions may be due to differing GC content and secondary structure [33]. The most efficient method of miRNA extraction from archived slides found here was the ‘isolation of FFPE tissue’ total RNA protocol, with an overnight Proteinase K digestion.

### Archived Slide Samples in Comparison to Fresh Bone Marrow

**Figure 3:**
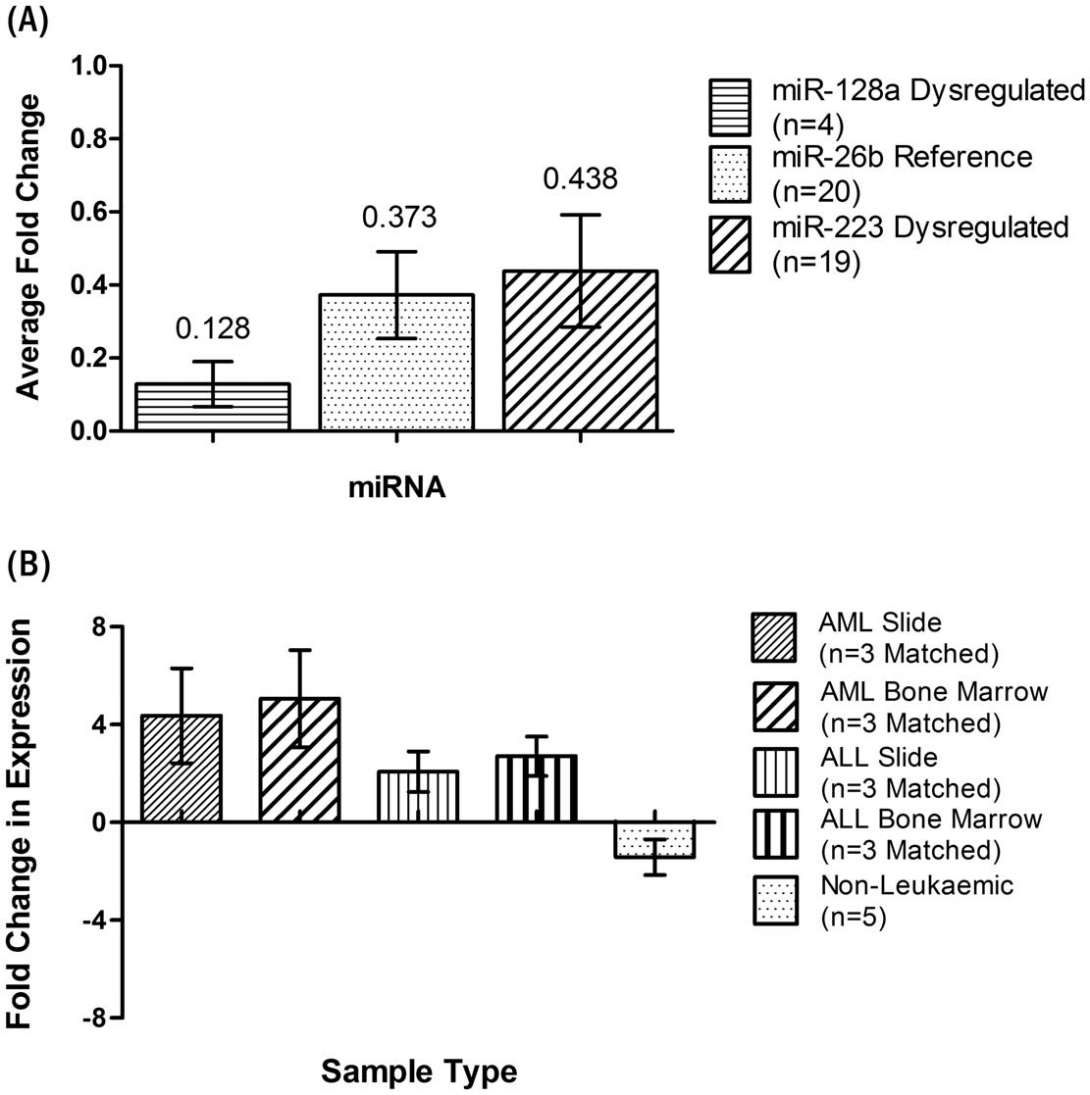
The average fold change in miRNA expression between matched patient fresh bone marrow aspirates and archived bone marrow slides. The average fold change in miRNA expression between matching time-point patient fresh bone marrow aspirates and archived slide bone marrow aspirates (Table S4). (**A**) **microRNA expression of matched patient fresh bone marrow and archived slide samples**. Three miRNA TaqMan assays were used (hsa-miR-128a, hsa-miR-26b, hsa-miR-223) and the Livak method for Fold Change analysis [34] to analyse the fold change difference between fresh Bone marrow and matching archived slides. hsa-miR-128a and hsa-miR-223 have been previously reported to have altered expression in leukaemia, whereas we have found here hsa-miR-26b is a stable reference. The overall difference in miRNA expression between fresh bone marrow samples and their matching archived slide as shown here is negligible. (**B**) **Difference in hsa-miR-223 expression in archived and fresh leukaemic samples**. Previously reported dysregulated miRNA hsa-miR-223 was used to analyse the fold change difference between leukaemic and non-leukaemic samples, to gauge the biological relevance of archived specimens in comparison to fresh. All samples were normalized to hsa-miR-26b. AML and ALL both showed fold change differences of hsa-miR-223 compared to non-leukaemic samples. The bone marrow and archived slides showed similar expression in both AML (Bone Marrow: 5.1±1.99 s.e.m.; Slide: 4.4±1.94 s.e.m.) and ALL (Bone Marrow: 2.71±0.81 s.e.m.; Slide: 2.1±0.83 s.e.m.).

The relationship in miRNA expression between matching archived slides and flash-frozen bone marrow samples was investigated to determine the utility of archived slides for expression analysis. Twenty fresh bone marrow and matched archived samples (Table S4) were analysed for hsa-miR-223, hsa-miR-128a and hsa-miR-26b expression. We found the average mean fold change in miRNA expression (using the Livak method [34]) between fresh and matched archived samples for all three miRNAs analysed to be minimal (Figure 3A, Average Fold Change: hsa-miR-128a 0.129±0.062 s.e.m.; hsa-miR-26b 0.373±0.12 s.e.m.; hsa-miR-223 0.438±0.154 s.e.m.). Similar results have been reported recently for miRNA expression analysis from FFPE sections [3], [21] and Core biopsies [18].

To confirm the biological relevance of material extracted from archived bone marrow smears, differential miRNA expression analysis of hsa-miR-223 and hsa-miR-26b was performed on leukaemic and non-leukaemic samples (Figure 3B; Table S4). Human hsa-miR-223 has been identified as crucial to myeloid regulatory networks [35], [36] and dysregulated in leukaemia [37], [38], [39]. Through our analysis here, we have identified hsa-miR-26b as a potential reference gene for future expression studies. Hsa-miR-26b has also been reported previously as a stable miRNA [27], [28]. Both AML and ALL bone marrow samples in this study showed substantial fold change differences in hsa-miR-223 expression compared to non-leukaemic samples. The average fold change in hsa-miR-223 expression between leukaemic and non-leukaemic ALL samples was 2.71 (±0.81 s.e.m.) from fresh bone marrow, and 2.07 (±0.83 s.e.m.) from archived slides. Similar expression differences were observed in AML samples with a fold change of 5.06 (±1.99 s.e.m.) from fresh bone marrow samples, and 4.36 (±1.94 s.e.m.) from archived slides, confirming observations from previous studies of hsa-miR-223 [38].

The differences we found in miRNA expression due to disease state were far greater than the differences between archived slides and their matching fresh bone marrow. However there are several limitations encountered during the course of this study, such as sample tissue and cellular heterogeneity (Table S3), as well as degradation of samples. The miRNA we have identified here as stable for certain hematopoietic studies have also previously been shown as differentially expressed in other tissues. For example, hsa-miR-26b has been implicated in carcinomas [40], [41], and hsa-miR-16 is reportedly dysregulated in Chronic Lymphoblastic Leukaemia patients [42], [43]. The careful selection of internal reference genes for miRNA normalization is therefore critical, and must be based on the sample types to be tested.

It has been proposed that alterations in miRNA expression play a critical role in the pathophysiology of many, perhaps all, human cancers [44]. Fresh frozen tissues are still seen as the gold standard for disease studies [2], however the demands for large cohorts and infinite resources means researchers are beginning to turn to the archives of pathology and histology departments. To realize this potential, development of proper methods of biomolecule extraction and analysis is warranted. In this regard FFPE samples for use in miRNA expression profiling have been thoroughly investigated, however the same cannot be said for archived slide smear samples. This study to our knowledge is the first to attempt miRNA expression analysis utilizing archived bone marrow aspirate slides. We have developed a protocol for isolation of total RNA from archival samples which includes an overnight Proteinase K digestion followed by a column based kit extraction. This method appears robust enough to allow the use of such samples in miRNA association studies for disease classification, diagnosis and prognosis.

## Materials and Methods

### Ethics Statement

This study was approved by the Royal Children’s Hospital (RCH) Ethics Committee (HREC reference #27027B).

### Samples

Samples used are existing archived fresh bone marrow specimens and bone marrow films taken during diagnosis, induction, remission and follow-up from paediatric acute leukaemia cases (Acute Lymphoblastic and Acute Myeloid) presented at the Royal Children’s Hospital, Victoria, Australia. This cohort consists of homogeneously treated patients with comprehensive clinical annotations.

The films used during this study are archived, air-dried, unstained bone marrow smear slides, with storage times varying from 13 years to six months. Matched patient fresh bone marrows (where used) were cryogenically frozen for no more than 5 years. All patients were <18 years of age. Control samples from bone marrow of unrelated and unaffected children were analysed in parallel, as well as multiple cell lines (K562 [45], DG-75 [46], NALM-6 [47], Jeg-3, BEL, REH [48], CCRF-CEM [49], BeWo [50], JWL, Jurkat [51], Kasumi-1 [52], THP-1 [53], MV-4-11 and AML-193 [54]).

### Extraction Methods

Before extraction, fresh bone marrow aspirates were processed using a Ficoll-Paque™ (GE Healthcare, Piscataway USA). The white cell layer was immediately cryo-frozen or stored in RNA*later*® (Ambion® by Life Technologies, Mulgrave, Victoria AUST) prior to extraction.

Both fresh bone marrow and archived slides were extracted using a TRIzol-based or kit-based method. The TRIzol® (Ambion®, catalogue #AM9738) method was used as per the manufacturer’s instructions. The Roche High Pure miRNA Isolation Kit (Catalogue #05080576001, Dee Why, NSW AUST) was used as per the manufacturer’s instructions where possible, however modifications to the protocol were needed to improve the total RNA yield (Table 1). After initial assessments, we chose to use the ‘miRNA isolation from Tissue’ and ‘miRNA isolation from FFPE’ kit extraction protocols (Table 1). The concentration and purity of all RNA samples were assessed using the NanoDrop® ND-1000 spectrophotometer (Thermo Fisher Scientific Inc., Scorsby, Victoria AUST). All RNA was stored at −80°C.

### Extraction Optimization Method

Nine patients (leukaemic and non-leukaemic) were used to evaluate the quality of 6 slide extraction methods (Table 1; Table S3). Six unstained archived slides, and two Giemsa-stained slides were obtained for each patient. To standardize the amount of starting material prior to extraction, the stained slides were cell counted as an approximate measure of cell density on the unstained slides. The volume of bone marrow added to each slide at creation is kept equivalent, and therefore the volume of marrow extracted from each slide was kept equivalent. Individual cell counts are listed in Table S3. The whole material was used from one glass slide from each patient to determine extraction efficiency.

Both the TRIzol and kit methods were modified to include a slide smear scraping step [12]. The cellular material was directly dissolved in TRIzol or lysis buffer on the glass slide and scraped using a Cell Scraper (Nunc®) into a 1.5 ml microcentrifuge tube prior to extraction.

### Quantitative Real-Time PCR

Expression analysis used Applied Biosystems TaqMan® microRNA Assays for reverse transcription and Real-Time PCR (qRT-PCR) (Applied Biosystems® by Life Technologies, Mulgrave, Victoria AUST). Amplification was measured by the Applied Biosystems 7300 Sequence Detection System. Each singleplex assay was performed according to the manufacturer’s instructions with all samples run in triplicate. Replicates were omitted if threshold cycle (C_t_) standard deviation greater than 1, or where C_t_ values were exactly 40. The threshold cycle (C_t_) is defined as the fractional cycle number at which the fluorescence passes a fixed threshold [55]. All cDNA was stored at −20°C and appropriate blanks were run.

The dynamic range and sensitivity of the TaqMan miRNA assays was tested by using synthetic oligonucleotides of known concentration to hsa-miR-16 and hsa-miR-26b to create a Standard Curve (Figure S1A). Multiple real-time PCR runs for both assays yielded consistent results for Range, Slope and R^2^ values, with a range of measurable values from 4.28×10^10^ molecules/µl (C_t_≈5) to 36.8 molecules/µl (C_t_≈40).

### Singleplex cDNA Reverse Transcription Conditions

Master Mix per sample: 0.15 µl 100 mM dNTPs, 1 µl MultiScribe Reverse Transcriptase, 1.5 µl 10× Reverse Transcription Buffer, 0.19 µl RNAse Inhibitor, 4.16 µl Nuclease-Free water, 3 µl Reverse Transcription Primer. 10 µl of Master Mix was added to a 0.2 ml microcentrifuge tube, then 5 µl of RNA extract. All samples were run on an Applied Biosystems 9700 Thermocycler with the following PCR conditions: 30 min at 16°C, 30 min at 42°C, 5 min at 85°C, Hold indefinitely at 4°C.

### Real Time PCR Conditions

Master Mix per sample: 10 µl TaqMan® 2× Universal PCR Master Mix, 7.67 µl Nuclease-Free water, 1 µl TaqMan® microRNA real-time assay primer. 18.67 µl of Master Mix was added to an Applied Biosystems 96 well optical plate, then 1.33 µl of reverse-transcribed RNA product. Real-time PCR conditions are as follows: 2 min at 50°C, 10 min at 95°C, 40 cycles of 15 sec at 95°C then 1 min at 60°C.

### Establishment of Reference Genes for Archived Bone Marrow Samples

To establish the optimal reference genes, 14 cell line samples were used and 27 archived patient bone marrow aspirate slides of both leukaemic (9 ALL and 9 AML) and non-leukaemic (n = 9) origin (Table S1). Each sample was measured using 14 RNA assays for qRT-PCR (Applied Biosystems® by Life Technologies). The 14 RNA assays tested are listed in Table S2. geNorm [25] and NormFinder [26] software packages were used to identify the most stably expressed genes within this set. All raw C_t_ values for each assay were first made linear and relative to each other using the ΔC_t_ method (outlined below, Equation 2) then input into the software packages. Reference genes were designated as ‘stable’ when their expression was most consistent across the tested sample types, disease states and extraction variables. The results obtained from geNorm and NormFinder were confirmed independently using DataAssist™ software (v.3.0 Applied Biosystems).

### Expression Analysis

All data was collected using Microsoft Excel and analysed using the LinRegPCR program v.11.0 [56]. All target and reference genes had PCR efficiencies nearing 100% (Efficiency measures >1.97), and each sample was within 5% of each other. All data was statistically analysed using Graph Pad PRISM® V.5 and confirmed using DataAssist™ software as above. The Livak Method [34] was used for Fold Change analysis based on the formulas below:

### Equation 1: Where a Target Gene was Measured in a Test Sample Relative to a Calibrator Sample, Normalized to the Expression of a Reference Gene



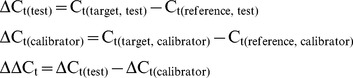


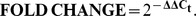



### Equation 2: Where the Effects of an Experimental Treatment were Measured on the Expression of a Candidate Gene, when only One Gene was Studied



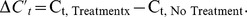


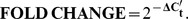



### Archived Slide Samples in Comparison to Fresh Bone Marrow

To evaluate archived bone marrow aspirate slides for miRNA expression, 20 unstained archived slides were chosen, with matching time-point fresh bone marrow available (Table S4). All samples were extracted using the Roche High Pure miRNA Isolation kit, ‘isolation from FFPE tissue’ Total RNA protocol with 3 hr Proteinase K digestion, which has been described here previously. Following extraction each sample was analysed using TaqMan miRNA assays hsa-miR-128a, hsa-miR-223 and hsa-miR-26b. The resulting data were corrected for PCR efficiency using LinRegPCR and analysed using the Relative quantitation methods above. Where slide sample miRNA expression was compared to its matching bone marrow (Figure 3A), Equation 2 was used without normalization to a reference gene. Where a miRNA expression difference was determined between diseased and non-diseased states (Figure 3B), Equation 1 was used with normalization to hsa-miR-26b.

To verify these results were reflecting the disease states, rather than due to differing Singleplex cDNA synthesis reactions, we undertook an additional Multiplex validation step using Applied Biosystems Megaplex™ Primer Pools (Human Pool A v2.1) following the manufacturers protocol. This Megaplex contains RT primers for simultaneous cDNA synthesis of 377 human miRNA from the one RNA sample; therefore target and reference miRNA can be analysed from the same RT reaction. This multiplex RT reaction then served as the starting material for independent qRT-PCR reactions (run on the same plate) using the miRNA and methods previously mentioned. Comparison of the qRT-PCR results for miRNA that were reverse transcribed as a group (Multiplex) verses those transcribed individually (Singleplex) showed no significant differences in qRT-PCR results (example: Figure S1B). Additionally using a subset of AML samples, we found no difference in the fold change results (leukaemic vs non-leukaemic hsa-miR-223 expression normalized to hsa-miR-26b) for Multiplex RT reactions as compared to Singleplex assays (Data not shown), as has also been reported previously [57], [58], [59].

## Supporting Information

Figure S1 (A) Dynamic Range and Sensitivity of TaqMan mature miRNA assays: Plotting multiple Standard curves of Synthetic Oligonucleotides of known quantity for Reference miRNA hsa-miR-16 and hsa-miR-26b. (B) Compatibility of Applied Biosystems mature miRNA Singleplex and Multiplex (utilizing Megaplex Human Primer Pool A v2.1) assays for downstream expression analysis.
(TIF)

Table S1 *Establishment of Reference Genes for Archived bone marrow samples:* Sample phenotype information for all samples utilized in this study.
(DOCX)

Table S2 RNA sequence, location and accession numbers for all utilized RNA assays.
(DOCX)

Table S3 *Optimization of miRNA Extraction from Archived bone marrow samples:* Phenotype information for all patients utilized in this study. Extraction Methods defined in Table 1.
(DOCX)

Table S4 *Archived slide samples in comparison to fresh bone marrow:* Sample phenotype information for all patients utilized in this study.
(DOCX)
