# Supplementary figures and images for: Evaluation of MicroRNA Expression in Patient Bone Marrow Aspirate Slides
Source: PLoS One. 2012 Aug 13;7(8):e42951. doi: 10.1371/journal.pone.0042951 (PMC3418238; doi:10.1371/journal.pone.0042951)

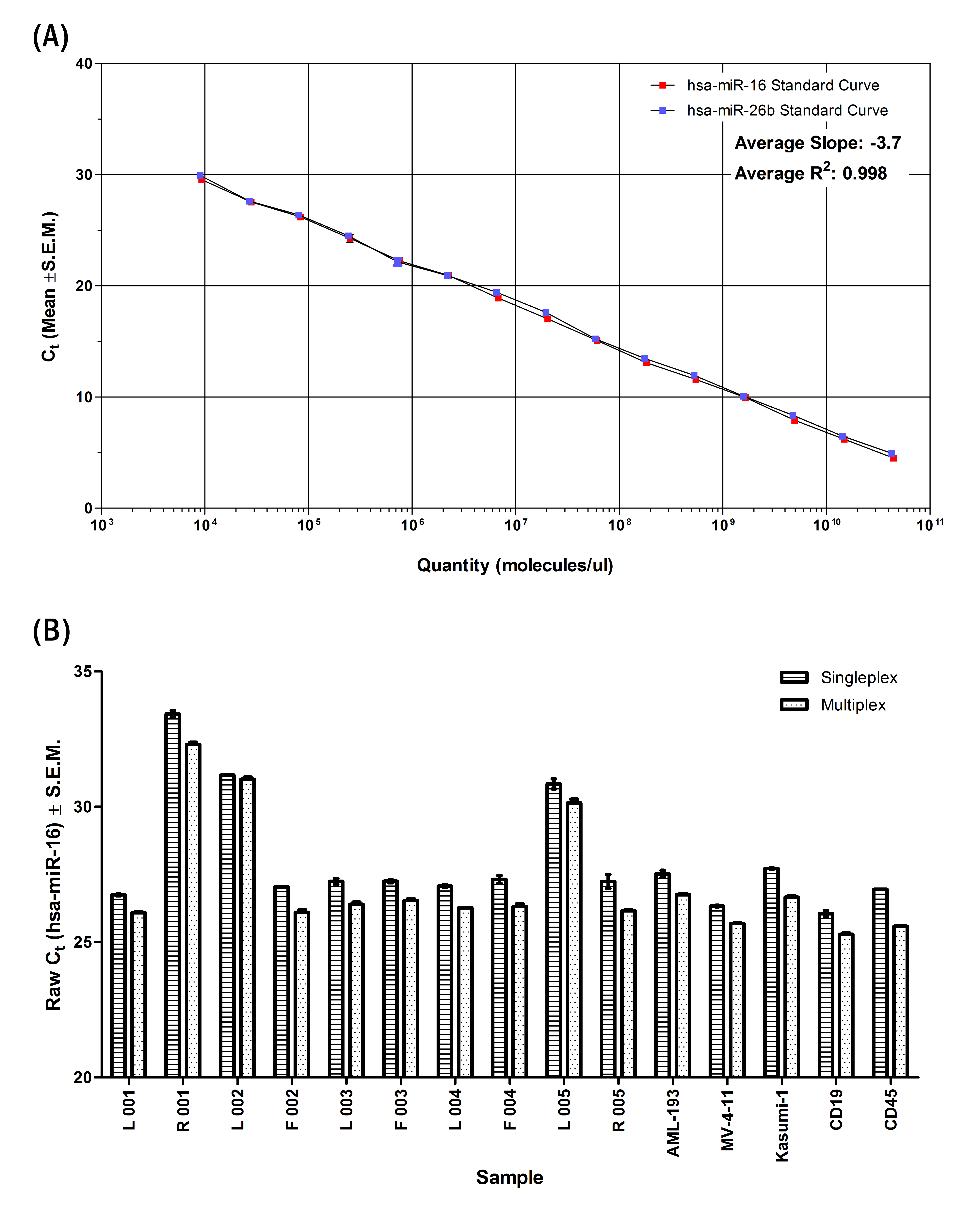

Supplement: Figure S1 — (A) Dynamic Range and Sensitivity of TaqMan mature miRNA assays: Plotting multiple Standard curves of Synthetic Oligonucleotides of known quantity for Reference miRNA hsa-miR-16 and hsa-miR-26b. (B) Compatibility of Applied Biosystems mature miRNA Singleplex and Multiplex (utilizing Megaplex Human Primer Pool A v2.1) assays for downstream expression analysis. (TIF) [file pone.0042951.s001.tif]
