# Supplementary material for: Evaluation of MicroRNA Expression in Patient Bone Marrow Aspirate Slides
Source: PLoS One. 2012 Aug 13;7(8):e42951. doi: 10.1371/journal.pone.0042951 (PMC3418238; doi:10.1371/journal.pone.0042951)
Supplement: Table S1 — Establishment of Reference Genes for Archived bone marrow samples: Sample phenotype information for all samples utilized in this study. (DOCX) [file pone.0042951.s002.docx]

| **ID** | **Type** | **Sex** | **Status** | **Diagnosis** | **NormFinder Group** |
| --- | --- | --- | --- | --- | --- |
| 1 | US Slide | M | Leukaemic | AML | Hematologic Cancer |
| 2-a | US Slide | F | Leukaemic | AML | Hematologic Cancer |
| 2-b | US Slide | F | Remission | AML | Non-Cancerous |
| 3-a | US Slide | M | Leukaemic | AML | Hematologic Cancer |
| 3-b | US Slide | M | Remission | AML | Non-Cancerous |
| 3-c | US Slide | M | Non-Leukaemic | AML | Non-Cancerous |
| 4-a | US Slide | F | Leukaemic | AML | Hematologic Cancer |
| 4-b | US Slide | F | Remission | AML | Non-Cancerous |
| 4-c | US Slide | F | Non-Leukaemic | AML | Non-Cancerous |
| 5 | US Slide | F | Leukaemic | AML | Hematologic Cancer |
| 6 | US Slide | M | Leukaemic | AML | Hematologic Cancer |
| 7 | US Slide | F | Leukaemic | AML | Hematologic Cancer |
| 8 | US Slide | F | Leukaemic | AML | Hematologic Cancer |
| 9 | US Slide | M | Leukaemic | AML | Hematologic Cancer |
| 10-a | US Slide | M | Leukaemic | ALL | Hematologic Cancer |
| 10-b | US Slide | M | Remission | ALL | Non-Cancerous |
| 11-a | US Slide | M | Leukaemic | ALL | Hematologic Cancer |
| 11-b | US Slide | M | Remission | ALL | Non-Cancerous |
| 12 | US Slide | M | Leukaemic | ALL | Hematologic Cancer |
| 13 | US Slide | F | Leukaemic | ALL | Hematologic Cancer |
| 14 | US Slide | M | Leukaemic | ALL | Hematologic Cancer |
| 15 | US Slide | M | Leukaemic | ALL | Hematologic Cancer |
| 16 | US Slide | M | Leukaemic | ALL | Hematologic Cancer |
| 17 | US Slide | M | Leukaemic | ALL | Hematologic Cancer |
| 18 | US Slide | M | Leukaemic | ALL | Hematologic Cancer |
| 19 | US Slide | F | Non-Leukaemic | Neuroblastoma | Non-Cancerous |
| 20 | US Slide | M | Non-Leukaemic | Normal | Non-Cancerous |
| MV-4-11 | Cell Line | M | Leukaemic | AML | Hematologic Cancer |
| AML-193 | Cell Line | F | Leukaemic | AML | Hematologic Cancer |
| THP-1 | Cell Line | M | Leukaemic | AML | Hematologic Cancer |
| Kasumi-1 | Cell Line | M | Leukaemic | AML | Hematologic Cancer |
| K562 | Cell Line | F | Leukaemic | CML | Hematologic Cancer |
| DG-75 | Cell Line | M | Cancerous | Burkitt’s Lymphoma | Hematologic Cancer |
| Nalm-6 | Cell Line | - | Leukaemic | Pre-B ALL | Hematologic Cancer |
| Jeg-3 | Cell Line | - | Cancerous | Choriocarcinoma | Non-Hematologic Cancer |
| BEL | Cell Line | - | Non-Leukaemic | Normal Lymphoblast | Non-Cancerous |
| REH | Cell Line | - | Leukaemic | ALL (non-T, non-B) | Hematologic Cancer |
| CCRF-CEM | Cell Line | F | Leukaemic | ALL | Hematologic Cancer |
| BeWo | Cell Line | M | Cancerous | Choriocarcinoma | Non-Hematologic Cancer |
| JWL | Cell Line | - | Non-Leukaemic | Normal Lymphoblast | Non-Cancerous |
| Jurkat | Cell Line | M | Leukaemic | Acute T-Cell Leukaemia | Hematologic Cancer |

US-Unstained; F-Female; M-Male; ALL-Acute Lymphoblastic Leukaemia, AML-Acute Myeloid Leukaemia
