# Supplementary material for: Evaluation of MicroRNA Expression in Patient Bone Marrow Aspirate Slides
Source: PLoS One. 2012 Aug 13;7(8):e42951. doi: 10.1371/journal.pone.0042951 (PMC3418238; doi:10.1371/journal.pone.0042951)
Supplement: Table S2 — RNA sequence, location and accession numbers for all utilized RNA assays. (DOCX) [file pone.0042951.s003.docx]

| **ID** | **Mature miRNA Sequence** | **Accession #** | **Chromosome Location** |
| --- | --- | --- | --- |
| *hsa-let-7b* | UGAGGUAGUAGGUUGUGUGGUU | miRBase: MIMAT0000063 | chr22: 46509571 - 46509592 |
| *hsa-miR-10a* | UACCCUGUAGAUCCGAAUUUGUG | miRBase: MIMAT0000253 | chr17: 46657221 - 46657243 |
| *hsa-miR-16* | UAGCAGCACGUAAAUAUUGGCG | miRBase: MIMAT0000069 | chr13:50623122-50623143 |
| *hsa-miR-26b* | UUCAAGUAAUUCAGGAUAGGU | miRBase: MIMAT0000083 | chr2:219267380-219267400 |
| *hsa-miR-34b* | CAAUCACUAACUCCACUGCCAU | miRBase: MIMAT0004676 | chr11: 111383712 - 111383733 |
| *hsa-miR-103a* | AGCAGCAUUGUACAGGGCUAUGA | miRBase: MIMAT0000101 | chr5: 167987948 - 167987970 |
| *hsa-miR-106a* | AAAAGUGCUUACAGUGCAGGUAG | miRBase: MIMAT0000103 | chrX: 133304240 - 133304262 |
| *hsa-miR-128a* | UCACAGUGAACCGGUCUCUUU | miRBase: MIMAT0000424 | chr2: 136423016-136423036 |
| *hsa-miR-186* | CAAAGAAUUCUCCUUUUGGGCU | miRBase: MIMAT0000456 | chr1:71533328-71533349 |
| *hsa-miR-223* | UGUCAGUUUGUCAAAUACCCCA | miRBase: MIMAT0000280 | chrX: 65238779 - 65238800 |
| *hsa-miR-361-5p* | UUAUCAGAAUCUCCAGGGGUAC | miRBase: MIMAT0000703 | chrX: 85158646 - 85158667 |
| *hsa-miR-374a* | UUAUAAUACAACCUGAUAAGUG | miRBase: MIMAT0000727 | chrX: 73507132 - 73507153 |
| *U6 snRNA* | GTGCTCGCTTCGGCAGCACATATACTAAAATTGGAACGATACAGAGAAGATTAGCATGGCCCCTGCGCAAGGATGACACGCAAATTCGTGAAGCGTTCCATATTTT | NCBI: NR_004394 | chr3:180949525-180949630 |
| *RNU44 (SNORD44)* | CCTGGATGATGATAGCAAATGCTGACTGAACATGAAGGTCTTAATTAGCTCTAACTGACT | NCBI: NR_002750 | chr1:173835106-173835166 |
