# Supplementary material for: Evaluation of MicroRNA Expression in Patient Bone Marrow Aspirate Slides
Source: PLoS One. 2012 Aug 13;7(8):e42951. doi: 10.1371/journal.pone.0042951 (PMC3418238; doi:10.1371/journal.pone.0042951)
Supplement: Table S3 — Optimization of miRNA Extraction from Archived bone marrow samples: Phenotype information for all patients utilized in this study. Extraction Methods defined in Table 1. (DOCX) [file pone.0042951.s004.docx]

| **ID** | **Type** | **Sex** | **Status** | **Slide Cell Count** | **Diagnosis** | **Extraction Methods (Table 1)** |
| --- | --- | --- | --- | --- | --- | --- |
| 1 | 6x US Slide | F | Leukaemic | 1,011,113 | ALL | 1-6 |
| 2 | 6x US Slide | F | Remission | 1,720,788 | AML | 1-6 |
| 3 | 6x US Slide | F | Remission | 200,116 | ALL | 1-6 |
| 4 | 6x US Slide | M | Remission | 799,953 | ALL | 1-6 |
| 5 | 6x US Slide | M | Leukaemic | 276,852 | ALL | 1-6 |
| 6 | 2x US Slide | F | Non-Leukaemic | 526,621 | AML | 5-6 |
| 7 | 2x US Slide | M | Remission | 547,686 | ALL | 5-6 |
| 8 | 4x US Slide | F | Remission | 449,403 | ALL | 1-4 |
| 9 | 2x US slide | M | Remission | 367,130 | ALL | 5-6 |

US-Unstained; F-Female; M-Male; ALL-Acute Lymphoblastic Leukaemia, AML-Acute Myeloid Leukaemia
