# Supplementary material for: Evaluation of MicroRNA Expression in Patient Bone Marrow Aspirate Slides
Source: PLoS One. 2012 Aug 13;7(8):e42951. doi: 10.1371/journal.pone.0042951 (PMC3418238; doi:10.1371/journal.pone.0042951)
Supplement: Table S4 — Archived slide samples in comparison to fresh bone marrow: Sample phenotype information for all patients utilized in this study. (DOCX) [file pone.0042951.s005.docx]

| **ID** | **Sample Type 1** | **Sample Type 2** | **Sex** | **Status** | **Diagnosis** | **Assays**  **(hsa-miR-)** |
| --- | --- | --- | --- | --- | --- | --- |
| ***1*** | ***US Slide*** | ***BM*** | ***M*** | ***Cancerous*** | ***ALL*** | ***128, 26, 223*** |
| ***2*** | ***US Slide*** | ***BM*** | ***F*** | ***Cancerous*** | ***AML*** | ***128, 26, 223*** |
| 3 | US Slide | BM | M | Cancerous | AML | 128, 26, 223 |
| ***4*** | ***US Slide*** | ***BM*** | ***F*** | ***Cancerous*** | ***AML*** | ***128, 26, 223*** |
| ***5*** | ***US Slide*** | ***BM*** | ***M*** | ***Cancerous*** | ***ALL*** | ***26, 223*** |
| ***6*** | ***US Slide*** | ***BM*** | ***M*** | ***Remission*** | ***ALL*** | ***26, 223*** |
| 7 | US Slide | BM | M | Non-Cancerous | AML | 26, 223 |
| ***8*** | ***US Slide*** | ***BM*** | ***F*** | ***Remission*** | ***ALL*** | ***26, 223*** |
| 9 | US Slide | BM | M | Remission | ALL | 26, 223 |
| 10 | US Slide | BM | M | Cancerous | ALL | 26, 223 |
| 11-a | US Slide | BM | F | Remission | AML | 26, 223 |
| 11-b | US Slide | BM | F | Remission | AML | 26, 223 |
| ***12*** | ***US Slide*** | ***BM*** | ***F*** | ***Remission*** | ***ALL*** | ***26, 223*** |
| ***13*** | ***US Slide*** | ***BM*** | ***M*** | ***Non-Cancerous*** | ***AML*** | ***26, 223*** |
| 14 | US Slide | BM | M | Remission | ALL | 26, 223 |
| ***15*** | ***US Slide*** | ***BM*** | ***M*** | ***Remission*** | ***ALL*** | ***26, 223*** |
| ***16*** | ***US Slide*** | ***BM*** | ***M*** | ***Cancerous*** | ***ALL*** | ***26, 223*** |
| ***17*** | ***US Slide*** | ***BM*** | ***F*** | ***Cancerous*** | ***AML*** | ***26, 223*** |
| 18 | US Slide | BM | M | Non-Cancerous | T-Cell Non-Hodgkin’s Lymphoma | 26, 223 |
| 19 | US Slide | BM | F | Remission | ALL | 26, 223 |

***Bold***: Samples used in ‘subset’ for hsa-miR-223 expression analysis.

US-Unstained; BM-Bone Marrow; F-Female; M-Male; ALL-Acute Lymphoblastic Leukaemia, AML-Acute Myeloid Leukaemia
